# Reduction of spontaneous cortical beta bursts in Parkinson’s disease is linked to symptom severity

**DOI:** 10.1093/braincomms/fcaa052

**Published:** 2020-04-28

**Authors:** Mikkel C Vinding, Panagiota Tsitsi, Josefine Waldthaler, Robert Oostenveld, Martin Ingvar, Per Svenningsson, Daniel Lundqvist

**Affiliations:** f1 Department of Clinical Neuroscience, NatMEG, Karolinska Institutet, 171 77 Stockholm, Sweden; f2 Department of Clinical Neuroscience, Neuro Svenningsson, Karolinska Institutet, Stockholm, Sweden; f3 Department of Neurology, University Hospital Marburg, Marburg, Germany; f4 Donders Institute for Brain, Cognition and Behaviour, Radboud University, Nijmegen, Netherlands; f5 Section of Neuroradiology, Karolinska University Hospital, Stockholm, Sweden

**Keywords:** beta band, beta bursts, bradykinesia, Parkinson’s disease, resting-state

## Abstract

Parkinson’s disease is characterized by a gradual loss of dopaminergic neurons, which is associated with altered neuronal activity in the beta-band (13–30 Hz). Assessing beta-band activity typically involves transforming the time-series to get the power of the signal in the frequency domain. Such transformation assumes that the time-series can be reduced to a combination of steady-state sine- and cosine waves. However, recent studies have suggested that this approach masks relevant biophysical features in the beta-band—for example, that the beta-band exhibits transient bursts of high-amplitude activity. In an exploratory study, we used magnetoencephalography to record beta-band activity from the sensorimotor cortex, to characterize how spontaneous cortical beta bursts manifest in Parkinson’s patients on and off dopaminergic medication, and compare this to matched healthy controls. We extracted the time-course of beta-band activity from the sensorimotor cortex and characterized bursts in the signal. We then compared the burst rate, duration, inter-burst interval and peak amplitude between the Parkinson’s patients and healthy controls. Our results show that Parkinson’s patients off medication had a 5–17% lower beta bursts rate compared to healthy controls, while both the duration and the amplitude of the bursts were the same for healthy controls and medicated state of the Parkinson’s patients. These data thus support the view that beta bursts are fundamental underlying features of beta-band activity, and show that changes in cortical beta-band power in Parkinson’s disease can be explained—primarily by changes in the underlying burst rate. Importantly, our results also revealed a relationship between beta burst rate and motor symptom severity in Parkinson’s disease: a lower burst rate scaled with increased severity of bradykinesia and postural/kinetic tremor. Beta burst rate might thus serve as a neuromarker for Parkinson’s disease that can help in the assessment of symptom severity in Parkinson’s disease or in the evaluation of treatment effectiveness.

## Teaser

We compared spontaneous beta bursts in recordings of sensorimotor activity from 3-min resting-state magnetoencephalography recorded in Parkinson’s patients and healthy controls. Parkinson’s patients showed an 11% reduction in the beta burst rate compared to controls. The duration and amplitude of beta bursts did not differ between groups.

## Graphical Abstract

**Graphical Abstract:**
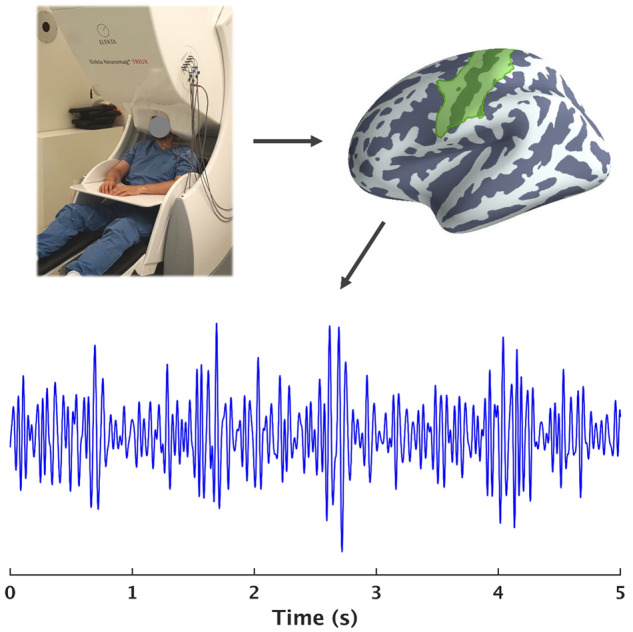

## Introduction

Parkinson’s disease is a neurodegenerative disease that, most often, initially diagnosed by the occurrence of motor symptoms tremor, rigidity and bradykinesia. The neurodegenerative process is characterized by a loss of dopamine and death of dopaminergic neurons throughout the basal ganglia–thalamic–cortical system (Rodriguez-Oroz *et al.*, 2009; Kalia and Lang, 2015). The dopamine loss leads to widespread functional changes in brain activity; for instance, throughout the basal ganglia–thalamic–cortical network, oscillatory activity in the beta band (13–30 Hz) exhibits systematic disease-related changes in Parkinson’s disease (Jenkinson and Brown, 2011). The direct influence of dopamine has for example been demonstrated to increase beta band power in the sub-thalamic nucleus (STN) when Parkinson’s patients are off dopaminergic medication as compared to on medication (Alonso-Frech *et al.*, 2006; Kühn *et al.*, 2006; Mallet *et al.*, 2008; Giannicola *et al.*, 2010; Neumann *et al.*, 2017). Increased beta power in the STN and the basal ganglia has further been linked to increased severity of bradykinesia and rigidity in Parkinson’s patients (Kühn *et al.*, 2006; Martin *et al.*, 2018). Disease-related changes in the beta band are found not only in STN and basal ganglia in Parkinson’s patients but are also present in the cortex, from where brain activity can be recorded non-invasively while patients are at rest, using magnetoencephalography (MEG) and electroencephalography (EEG).

Studies using MEG to assess neural activity while the participants were at rest show that Parkinson’s patients have decreased cortical beta power compared to healthy controls (Bosboom *et al.*, 2006; Heinrichs-Graham *et al.*, 2014). However, in the early stages of Parkinson’s disease, there seems to be an increase in beta power at rest compared to healthy controls (Pollok *et al.*, 2012). Treatments for Parkinson’s disease also seem to be effective through modulation of the cortical beta activity. Administration of dopaminergic medication increases the cortical beta power in Parkinson’s patients (Heinrichs-Graham *et al.*, 2014; Melgari *et al.*, 2014). Similarly, Parkinson’s patients treated with electrical deep brain stimulation showed an increase in cortical sensorimotor beta power following deep brain stimulation compared to off treatment (Airaksinen *et al.*, 2012; Cao *et al.*, 2017). However, other studies have reported that deep brain stimulation leads to a broader suppression of 5–25 Hz power in frontal and sensorimotor cortex (Abbasi *et al.*, 2018; Luoma *et al.*, 2018).

It is currently unclear whether the different directions of these disparate findings are due to differences in the Parkinson’s patients cohorts (e.g. early-stage versus later-stage Parkinson’s disease) or if they are due to uncertainties in the methods used to quantify beta activity. Beta activity is traditionally assessed by analysing the MEG/EEG data in the frequency-domain, using various forms of Fourier-transforms (e.g. wavelet-analysis) of the data. Fourier-based methods assume that the oscillatory activity in the time-series can be resolved as a sum of steady-state sine and cosine waves of varying frequency. There is, however, converging evidence that the oscillatory activity in the beta band does not occur at a steady state but instead consists of short transient bursts lasting only one to a few beta band cycles (Leventhal *et al.*, 2012; Bartolo and Merchant, 2015; Feingold *et al.*, 2015; Sherman *et al.*, 2016). From the resulting power spectral densities (PSD) it is impossible to tell whether changes in beta band reflect a general change in the amplitude of steady-state oscillations, or if it reflects changes in the occurrence or amplitude of transient beta bursts. In all three cases, the output from the Fourier-transform will sum up to a shift in beta band power.

Several recent studies have explored the functional role of transient beta bursts in the somatosensory cortex of healthy subjects. For instance, Shin *et al.* (2017) showed that the detection rate of a tactile stimulation was higher when the probability of a beta burst immediately before the stimulation was low, suggesting that the beta bursts exhibit a transient inhibitory effect on the processing of incoming sensory signals. The negative relationship between the probability of a beta burst and the detection rate of tactile stimulation were consistent in mice, monkeys and humans (Sherman *et al.*, 2016; Shin *et al.*, 2017). Similarly, Little *et al.* (2019) showed a negative relationship between the probability of cortical beta bursts before a cued movement and reaction time in a cued reaction task, demonstrating that beta bursts have an inhibitory effect on outgoing movement initiation. Assessment of changes in beta activity in terms of transient bursts—rather than averaging in the frequency domain—may contribute to a better understanding of what aspect of the beta-band activity changes in Parkinson’s disease due to disease and medication.

There is similar evidence on the functional role of transient beta bursts from research assessing beta-band activity in midbrain structures. The overall power changes in the beta band in the STN can, for example, be explained as changes in the occurrence of short periods with high beta amplitude (Tinkhauser *et al.*, 2017*a*, 2018). The high-amplitude beta bursts in STN showed both increased rate and longer duration*s* when the patients were off dopaminergic medication than on medication. Lofredi *et al.* (2019) used similar measurements from STN in patients undergoing surgery to find a decrease in beta bursts in the period leading up to a movement in a cued reaction task. The relation between beta bursts and movement initiation makes beta burst a potential tool for understanding loss of control and slowing of movement in Parkinson’s disease (Tinkhauser *et al.*, 2017*b*; Lofredi *et al.*, 2019).

Analysis of beta activity at the level of beta bursts appear to be a functionally relevant approach for further understanding sensory-motor processing and may provide new insights into the function of the sensory-motor system that is lost in average-based analysis method. Assessment of spontaneous beta bursts in Parkinson’s patients from non-invasive recordings, such as MEG might provide a more sensitive assessment on how the beta band activity changes due to the disease and may help to resolve the apparently conflicting results that emerge when assuming beta-band activity consist of steady-state beta oscillations.

In this study, we used non-invasive MEG measurements from Parkinson’s patients off and on dopaminergic medication, and measurements from matched healthy controls, to investigate the occurrence of spontaneous transient beta bursts in the sensorimotor cortex. Our primary aim was to compare the characteristics (such as duration, amplitude and rate) of spontaneous beta burst in the sensorimotor cortex of Parkinson’s patients to healthy controls. Our secondary aim was to explore whether any of the beta bursts characteristics changed with the presence of dopaminergic medication. Finally, a third aim was to investigate whether any of the beta bursts characteristics were linked to the severity of disease symptoms in Parkinson’s disease.

## Materials and methods

### Participants

Twenty patients diagnosed with Parkinson’s disease (age 41–85; five female) and 20 healthy controls (age 54–76; eight female) participated in the study. The study was approved by the regional ethics committee (Etikprövningsnämden Stockholm, DNR: 2016/911-31/1) and followed the Declaration of Helsinki. All participants gave written informed consent before participating.

The patients were recruited from the Parkinson’s Outpatient Clinic, Department of Neurology, Karolinska University Hospital, Stockholm, Sweden. The inclusion criteria for the Parkinson’s patients were a diagnosis of idiopathic Parkinson’s disease according to the UK Parkinson’s Disease Society Brain Bank Diagnostic Criteria with Hoehn and Yahr stages 1–3 (Hoehn and Yahr, 1967), under treatment with Levodopa, Catechol-O-methyltransferase inhibitor (COMT) inhibitors, Monoaminoxidase-B (MAO-B) inhibitors or dopamine receptor agonists. Besides the diagnosis of Parkinson’s disease, the patients were healthy according to a physical and neurological examination.

Healthy controls were recruited among healthy participants who previously had participated in studies within the preceding year, or amongst the patients’ spouses.

Exclusion criteria for both groups were a diagnosis of major depression, dementia, history or presence of schizophrenia, bipolar disorder, epilepsy or history of alcoholism or drug addiction according to the *Diagnostic and Statistical Manual of Mental Disorders DSM-V* (American Psychiatric Association, 2013). Additional exclusion criteria for the healthy controls were a diagnosis of Parkinson’s disease or any form of movement disorder.

One patient cancelled the participation in the study due to severe tremor in the non-medicated state. One healthy control only completed one session and was not included in the analysis. The analysis includes 19 patients and 19 healthy controls.

### Procedure

The patients were instructed to omit their morning dose of dopaminergic medication on the day of participation. Thus, the non-medicated state was defined as a withdrawal period of 12 h after the last dopaminergic medication. Patients were further instructed to bring their prescribed dose of medication, which they had to take during the experiment. All patients followed the instructions.

Preparation for the MEG recordings began as soon as the participants were briefed about the procedure and signed the written informed consent. The recordings consisted of 3 min where the participants sat with their eyes closed in the MEG scanner. Text on a screen placed in front the participants initially instructed the participants to close their eyes. Participants were instructed not to open their eyes before being told to, and to avoid moving until they were allowed to open their eyes. The recordings began once the experimenter through video observation had assured that participant’s eyes were closed. The participants then did two unrelated tasks in the same recording session consisting of an active tapping task and a task with passive movements (Vinding *et al.*, 2019). Each MEG recording session took about1 h.

When the first session was over, participants had a break outside the scanner. During the break, the participants performed the neurological tests described below, and the patients took medication. The second MEG measurement began ∼1 h after medication. The healthy controls were measured twice with a 1-h break in-between to accommodate the potential effect of the fixed order of the off/on medication measurements in patients.

Motor function was assessed in all participants using the motor subscale of the Movement Disorder Society’s Unified Parkinson’s Disease Rating Scale (MDS-UPDRS-III) (Goetz *et al.*, 2007), by neurologists certified in the use of MDS-UPDRS. Patients were assessed immediately after the first MEG session in the non-medicated state and again after the second MEG session on medication. Montreal Cognitive Assessment (MoCA) test was done on medication.

### MEG recordings

MEG data were recorded with an Elekta Neuromag TRIUX 306-channel MEG system, with 102 magnetometers and 102 pairs of orthogonal planar gradiometers, inside a two-layer magnetically shielded room (model Ak3B, Vacuumschmelze GmbH), with internal active shielding active to suppress electromagnetic artefacts. Data were recorded at 1000 Hz with an online 0.1 Hz high-pass filter and 330 Hz low-pass filter. The subjects’ positions and movements inside the MEG scanner were measured during recordings with head-position indicator coils attached to subjects’ heads. The location of the coils—and additional points giving a representation of the subjects’ head shape—was digitalized with a Polhemus Fastrak motion tracker before the measurements. The head shapes were later used to co-register MEG data and structural MRI. Horizontal and vertical electrooculogram and electrocardiogram were recorded simultaneously with the MEG.

### Data processing

MEG data were processed off-line by applying temporal signal space separation to suppress artefacts from outside the scanner helmet and correct for head movement during the recordings (Taulu and Simola, 2006). The temporal signal space separation had a buffer length of 10 s and a cut-off correlation coefficient of 0.95. Movement correction was done by shifting the head position to a position based on the median head position during the recording. We then did an independent component analysis for each subject using the *fastica* algorithm (Hyvarinen, 1999) implemented in MNE-Python (Gramfort *et al.*, 2013) in Python 2.7. Components related to saccadic eye-movements and heartbeats were identified based on their correlation with the electrooculogram or electrocardiogram and removed from the data.

We then applied source reconstruction to the data using noise weighted minimum-norm estimates (dSPM) (Dale *et al.*, 2000). The noise covariance matrix was estimated from 2 min of empty room data recorded before each session. The source space consisted of 5124 evenly spaced points sampled across the white matter surfaces. The surfaces were obtained with the automatic routine for extracting cortical surfaces in Freesurfer (Dale *et al.*, 1999) from individual T1 weighted MRI that were obtained on a GE Discovery 3.0 T or a Siemens Prisma 3.0 T MR scanner. One subject did not complete an MR scan, so we used an MRI template (Holmes *et al.*, 1998) warped to the subject’s head shape as a substitute. From the MRI, we obtained the inner skull boundary, which was used to create a single compartment volume conductor model to estimate the forward model.

**Figure 1:**
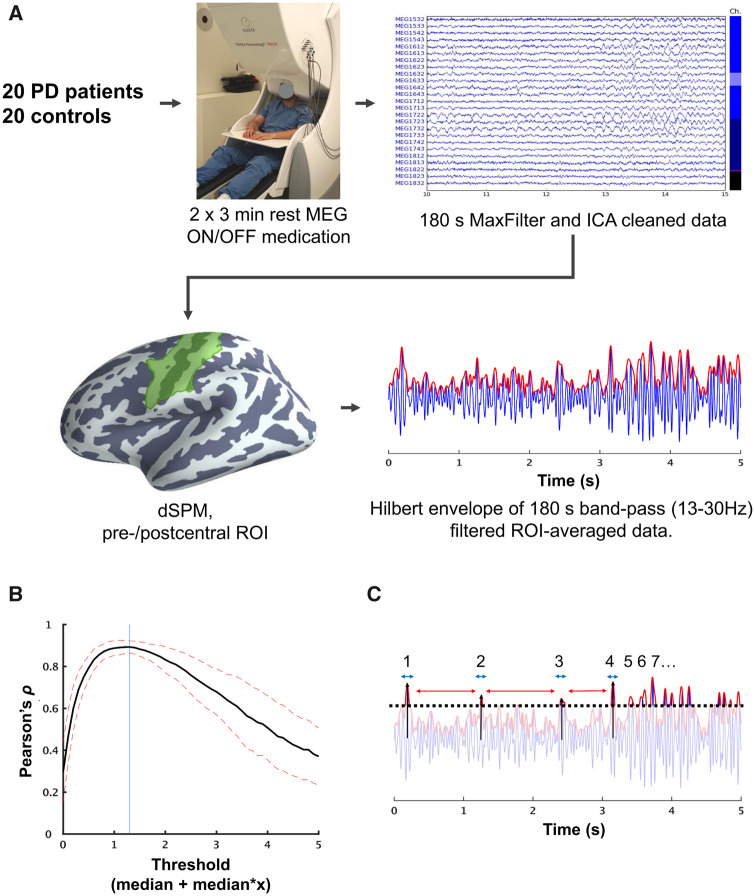
**Overview of data processing from raw MEG data to characterising beta bursts.** (**A**) We recorded 3 min of resting-state MEG. The raw MEG data were first processed with temporal signal space separation and independent component analysis to remove artefacts. We then did a dSPM source reconstruction and extracted the time-series from an ROI consisting of the pre-/post-central gyri and central sulcus. The ROI time-series was filtered to the beta range (13–30 Hz) and Hilbert-transformed. (**B**) Beta bursts were determined based on a threshold defined as the cut-off that had the highest correlation between the number of bursts and the mean amplitude in consecutive 3.0 s segments. The vertical line indicates the threshold used in the analysis. (**C**) Once the threshold was defined, we compared four features of the beta bursts: rate, duration (blue arrow), the inter-burst interval (red arrow) and peak amplitude (black arrow). PD = Parkinson’s disease; MEG = magnetoencephalography; ICA = independent component analysis; dSPM = dynamic statistical parametric mapping; ROI = region of interest.

The cortical surface was then segmented into anatomical labels based on the automatic labelling algorithm in Freesurfer (Destrieux *et al.*, 2010). Based on the labels, we extracted data from all point within a region of interest (ROI) consisting of the pre- and post-central gyri and central sulcus of the left hemisphere (Fig. 1). We then obtained a combined ROI time course as the first right-singular vector of a singular value decomposition of the source time courses within the ROI, with the sign of the vector normalized relative to the source orientations.

The ROI time-series was band-pass filtered between 13 and 30 Hz using a zero-phase finite impulse response filter to get the beta band time-course. The filter had a transition bandwidth of 3.25 Hz for the lower pass-band edge and a transition bandwidth of 7.5 Hz for the upper edge. We then applied a Hilbert transformation to the filtered time-series to obtain the instantaneous beta power.

### Defining beta bursts

To assess and compare beta burst, we defined high-amplitude bursts in the envelope of the time-series above a fixed threshold defined in order of medians above the median of the envelope for each participant. To determine the value of the threshold, we took the correlation coefficient between the average amplitude of the signal envelope and the number of bursts within consecutive 3.0 s of data. This gave a single correlation coefficient per threshold per subject, which were averaged across all subjects. The threshold with the highest correlation was used as the fixed threshold in the comparisons (Fig. 1B). Defining the threshold in orders of medians, rather than an absolute cut-off value, gives a threshold that preserved the statistical properties at the group-level but fitted to the dynamic range of the individual subjects’ time-series. Similar methods for defining thresholds have been used to identify beta bursts in event-related studies (Feingold *et al.*, 2015; Shin *et al.*, 2017). Here we extended the method to resting-state MEG.

Once the threshold was defined, we extracted four features of the beta bursts (Fig. 1C). The first feature was the rate of occurrence within the 3-min time-series. The purpose of the first feature was to answer if the beta band were more ‘bursty’ in one group compared to the other and whether it changed due to medication. The second feature was the burst *duration*, defined as the time between the burst reached the half-max of the peak value until it once again reached the half-max of the peak value. The purpose of the second feature was to answer if the beta bursts resembled ‘true’ bursts, i.e. with durations approximating one or two beta cycles or perhaps showed prolonged high-amplitude activity in one of the groups. The third feature was the *inter-burst interval*, defined as the time from the offset of one burst to the onset of the next. The fourth and final feature was the *peak amplitude* of the envelope within each burst.

### Power spectral densities

To compare how the time-domain analysis compares to Fourier-based analysis of beta power in the frequency domain, we calculated the PSD of the unfiltered ROI time-series in the spectrum from 1 to 48 Hz. We divided the time-series into consecutive epochs of 3 s with a 50% overlap and applied a Hanning taper before applying a fast Fourier transform using FieldTrip (Oostenveld *et al.*, 2011) in MATLAB (R2016b; MathWorks Inc.).

To quantify and compare the PSD between groups and across sessions we quantified the relative beta power, the 1/f broadband characteristic of the PSD and the beta power with the beta band, with the 1/f broadband characteristic removed. The relative power was calculated by integrating the PSD in the beta range (13–30 Hz) and dividing it by integral of the full spectrum.

PSDs of neural signals tend to follow a 1/f distribution where the PSD is approximately linear in log–log space, except for peaks usually found at the alpha or beta bands. By regressing the 1/f broadband characteristics out, one can yield an estimate of band-specific power that is unbiased by differences in broadband characteristics. For this purpose, we used the *fitting oscillations & one over f* (FOOOF) toolbox (Haller *et al.*, 2018) to analyse the broadband characteristics and calculate the beta band power with the 1/f broadband characteristics removed. In short, the procedure consists of an iterative linear regression to the full log-transformed spectra. The log-linear function is then subtracted from the data, and Gaussian functions are then fitted to the peaks in the remaining spectra in acceding order starting with the largest peak in the PSD. The centre of the Gaussians will then corresponded to the centre frequency of the peak and the height to the power of the peak. The Gaussian fits are then subtracted from the PSD, and a new log-linear function is fitted to the PSD. The log-linear function and Gaussian functions are then combined to assess the goodness of fit. For the fitting set a restriction to fit a maximum of eight Gaussian functions to avoid overfitting.

We ran this procedure on the PSD for each participant to find the parameters of the linear function fitted to the broadband spectra, to compare the 1/f characteristics and to assess the power of the beta band *without* the contribution of the 1/f broadband characteristics.

### Statistical analysis

#### Group characteristics

First, we tested for differences in age, sex ratio and MoCA score between the Parkinson’s patients and healthy controls to ensure that the demographics of the two groups were matched. Comparison of age and MoCA score by ‘Bayesian *t*-tests’ (Rouder *et al.*, 2009) using the *BayesFactor* package (Morey and Rouder, 2018) for R (R Core Team, 2013). The test gives the ratio of evidence for the hypothesis that there is a group difference versus the null hypothesis of no difference between groups. To test for difference in the male–female ratio between groups, we used a Bayesian test for unequal multinomial distributions (Gûnel and Dickey, 1974).

#### Power spectral densities

The statistical comparison of the relative beta power was done by pairwise Bayesian *t*-tests with the *BayesFactor* package in R. The same test was done for the beta power subtracted the 1/f broadband characteristics estimated from the combined 1/f and Gaussian regression model and the estimated peak frequency. For the analysis of 1/f and oscillatory features of the PSD, we compared the intercept and slope of the linear fit to the 1/f broadband characteristic between the patient and control groups and across sessions with pairwise Bayesian *t*-tests.

#### Beta burst features

The burst rate, burst duration, inter-burst interval and peak amplitudewere all analysed by Bayesian mixed-effect regression, estimated in R with the *brms* package (Bürkner, 2017). The models used uninformative priors and were estimated by Markov-Chain Monte-Carlo sampling drawing 20 000 samples across four chains and discarding the first half of each chain. The convergence of the chains was confirmed by checking R̂ ≈ 1 (Gelman and Rubin, 1992).

We analysed the *burst rate* by mixed-effect Poisson regression containing Group (patient/control) and Session (first/second) as fixed effects with subjects as a random effect. The analysis of *burst duration*, *inter-burst interval* and *peak amplitude* used the respective values for each burst by modelling the value of the *i*th burst for participant *j* as a function of Group and Session by mixed-effect regression. The *inter-burst interval* model used a lognormal link function. The models for *burst duration* and *peak amplitude* used shifted lognormal link functions.

Since the study was explorative, i.e. we did not have a clear hypothesis about how or if there would be differences in the quantifications of beta bursts, we aimed for a Bayesian statistical approach that does not rely on testing if there is a distinction between groups/session, but provide evidence for or against differences. Comparison between groups and sessions was done by comparing the marginal evidence—or Bayes factor (BF)—between models with and without the factors Group, Session and the interaction Group × Session as fixed effects. BF > 1 is evidence for the alternative hypothesis, whereas BF < 1 is evidence for the null hypothesis. We use the nomenclature by Wetzels *et al.* (2011) on the strength of the evidence where BF > 3 and BF < 0.33 is taken as conclusive support for the, respectively, alternative or null hypnosis. Values between 0.33 and 3 are inconclusive evidence. Post hoc hypothesis testing was done by determining if at least 95% posterior distribution of individual parameters did not contain zero. The resulting test statistic is the probability *P* ranging from 0 to 1. *P* close to 0 is evidence for a difference between conditions, whereas *P* close to 1 provides evidence against a difference. We used the 95% posterior distribution corresponding to critical alpha = 0.05.

#### Sensitivity and specificity analysis

In addition to the inferential statistical comparison between groups, we did a sensitivity/specificity analysis to assess how well the various beta band features could determine if a participant belonged to the patient or control group. For the features of beta bursts (burst rate, inter-burst interval, duration and amplitude), and the quantifications of the PSD (relative beta power, beta peak power and the slope and intercept of the 1/f log-linear regression), we fitted logistic regression models with the different quantitative measurements as predictor and the group as outcome variable. From the logistic models, we then calculated the area under the receiver operating characteristic (ROC) curve and the value of the optimal threshold.

#### Comparison across thresholds

To explore if the inference from the primary analysis was dependent on the threshold used to define bursts in the signal, we repeated the comparison of the burst rate between groups and sessions across thresholds. At each threshold—starting at the median to five times the order of median in steps of 0.1—we defined bursts as described above. The number of beta bursts at each threshold was analysed by mixed-effect Poisson regression as in the primary analysis. We then compared models with and without the factor Group, Session and the interaction between Group and Session to get BFs for each factor at each threshold. The model used uninformative priors and was estimated by Markov-Chain Monte-Carlo sampling drawing 4000 samples across four chains and discarding the first half of each chain.

Similar as described above, we calculated the ROC curves across thresholds to test how the sensitivity/specificity analysis to differentiate between the patient and control groups were dependent on the threshold used to define beta bursts.

#### Beta burst rate and motor symptoms

In addition to the group-level comparisons, we investigated the relationship between the burst rate and motor symptom severity measured with MDS-UPDRS-III for the Parkinson’s patients. Since previous studies have shown that beta-band PSD is correlated with specific motor symptoms of rigidity and bradykinesia (Airaksinen *et al.*, 2012, 2015; Melgari *et al.*, 2014), we divided the MDS-UPDRS-III scores into six subscales of different motor symptoms according to the factors described by Goetz *et al.* (2008) with the exception that left- and right-side bradykinesia was combined into one factor. Each MDS-UPDRS-III factor (midline function, rest tremor, rigidity, bradykinesia, postural and kinetic tremor, lower limb bradykinesia) was modelled by mixed-effect Poisson regression as a linear function of the burst rate with subject and session as random intercepts. With these models, we tested the association between beta burst rate and the MDS-UPDRS-III factor scores by testing if at least 95% of the posterior distribution did not contain zero. All models used uninformative priors and was estimated with *brms* (Bürkner, 2017) by Markov-Chain Monte-Carlo sampling drawing 20 000 samples across four chains and discarding the first half of each chain.

### Data availability

The datasets collected for the current study contains patient information that cannot be made public. The dataset is available from the corresponding author for review purpose or on reasonable request. Scripts for running the analysis presented in the paper are available at www.github.com/mcvinding/PD_beta_bursts.

## Results

### Group characteristics

The groups are adequately matched for comparison, as there were no systematic differences in the demographic variables: male/female ratio (BF = 0.60), age (BF = 0.41), and cognitive ability (BF = 0.39), see Table 1. The Parkinson’s patients showed 26–72% (mean 49%) reduction of motor symptoms on the MDS-UPDRS-III in the medicated state compared to the non-medicated state (BF = 4.70 × 10^7^).

**Table 1:** Summary of the Parkinson’s group and control group

	Parkinson’s patients	Healthy controls
*N*	19	19
Sex	5 females, 14 males	8 females, 11 males
Age	44–85 years (mean: 67.3 years)	54–76 years (mean: 69.3 years)
Disease duration	1–14 years (median: 4.5 years)	
LEDD	300–1150 mg (median: 615 mg)	
MDS-UPDRS-III (non-medicated)	10–61 (median: 34)	
MDS-UPDRS-III (medicated)	5–39 (median: 16)	
MoCA	25.5 (SD: 2.9)	26.1 (SD: 1.8)

LEDD = levodopa equivalent daily dosage; MDS-UPDRS-III = Movement Disorder Society’s Unified Parkinson’s Disease Rating Scale part III; MoCA = Montreal Cognitive Assessment.

### Power spectral densities

**Figure 2:**
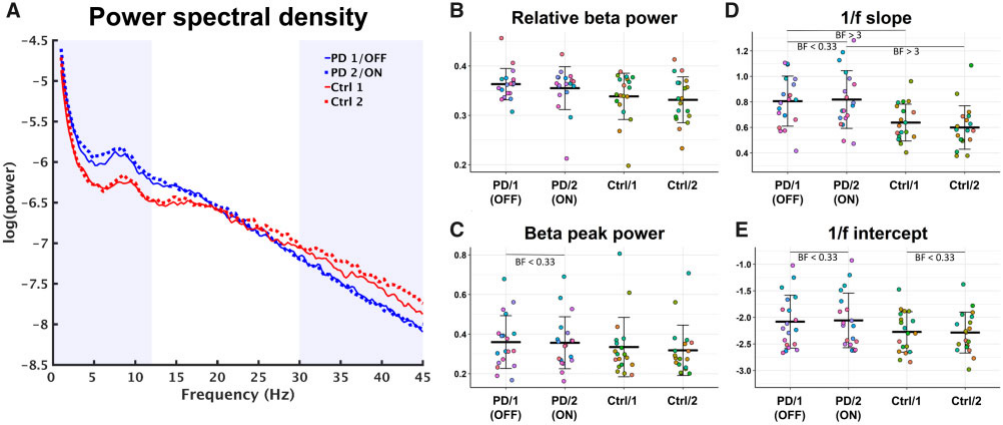
**Characteristics of the PSD.** (**A**) PSD for Parkinson’s patients (blue) and healthy controls (red). Solid lines are the first session/non-medicated and dashed lines are the second session/medicated. (**B**) The relative power of the beta band (13–30 Hz) across groups and sessions. (**C**) The power of the largest peak in the beta band relative to the 1/f broadband PSD across group and session. (**D**) The slope coefficient for each participant of the log-linear regression of the broadband PSD across group and session. (**E**) The intercept of for each participant of the log-linear regression of the broadband PSD across group and session. PD = Parkinson’s disease; Ctrl = healthy controls; BF = Bayes factor.

Comparison of the relative beta power of the sensorimotor ROI time-series (Table 2 and Fig. 2B) gave evidence against a difference between the first and second session for the controls (BF = 0.37) and against a difference between on/off medication for the Parkinson’s patients (BF = 0.34). On the other hand, the comparison between the groups in the first session/non-medicated state showed evidence for a difference between the groups but only as inconclusive evidence (BF = 1.27) and gave inconclusive evidence against a difference between the groups in the second session (BF = 0.87).

**Table 2:** Summary of PSD features across groups and sessions (mean + SD)

Group-session	Relative beta power	Broadband 1/f regression		Beta	
		Intercept	Slope	Peak freq.	Peak power
Parkinson’s patients 1/non-medicated	0.34 (0.047)	−2.08 (0.50)	0.81 (0.20)	18.5 (4.2)	0.36 (0.13)
Parkinson’s patients 2/medicated	0.33 (0.046)	−2.06 (0.51)	0.82 (0.23)	18.7 (3.4)	0.36 (0.13)
Healthy controls 1	0.36 (0.032)	−2.27 (0.38)	0.64 (0.14)	19.2 (3.0)	0.33 (0.15)
Healthy controls 2	0.36 (0.043)	−2.29 (0.38)	0.60 (0.17)	19.9 (4.3)	0.32 (0.13)

Relative beta power as the area under the curve in the beta band divided by the area under the curve of the entire spectrum. Intercept and slope of the linear fit to the 1/f characteristics of the broadband PSD. Beta PSD peaks frequency and peak power in the beta band estimated by subtracting the contribution of the 1/f log-linear fit.

All participants showed peaks in the beta band after removing the 1/f characteristics of the broadband PSD—Table 2 show summaries of the peak frequency and peak power across groups and session. The pairwise comparisons of the peak frequency all gave evidence against group differences in the peak beta PSD frequency in the first session (BF = 0.32) and seconds session (BF = 0.35), as well as within-group between sessions (BF = 0.30 for patients and BF = 0.39 for controls). A similar analysis for alpha and theta band is presented in the supplementary material. The between-group comparison of the beta PSD peak power (Fig. 2C) gave evidence against differences in both the first session (BF = 0.35) and second session (BF = 0.43) and for the comparisons within groups (BF = 0.24 for patients and BF = 0.40 for controls).

Table 2 and Fig. 2D–E show the model parameters for the linear fit to the broadband 1/f characteristics. As hinted in the averaged PSD (Fig. 2A), there were differences in the slope of the log-linear 1/f regression (Fig. 2D) between patients and healthy controls in both the first (BF = 8.9) and second session (BF = 19.6). There was, however, evidence against differences across sessions for both the patient group (BF = 0.27) and control group (BF = 0.62). The comparisons of 1/f intercept (Fig. 2E) favoured no differences between groups in the first session (BF = 0.63) and second session (BF = 0.80), though both BFs are in the conclusive range. For the within-group comparison, there were evidence against a difference in intercept for both (BF = 0.26) patients and controls (BF = 0.25).

### Beta burst rate

**Figure 3:**
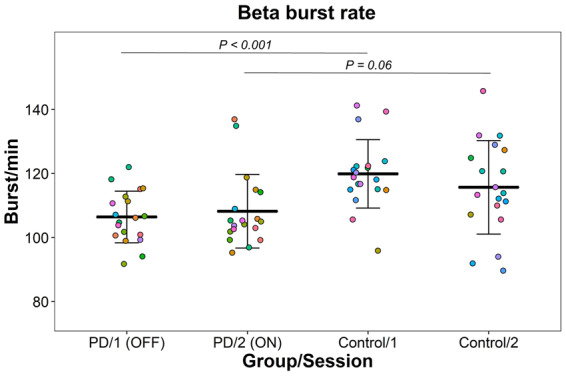
**Beta burst rate in the sensorimotor cortex across groups and sessions.** The points represent the beta bursts rate for each participant. The bars are means and standard deviations.

The Parkinson’s patients showed an average rate of 106 bursts/min (SD: 8) in the first session/non-medicated and 108 bursts/min (SD: 11) in the second session/medicated. The controls had an average rate of 120 bursts/min (SD: 11) in the first session and 116 bursts/min (SD: 15) in the second session. Figure 3 shows the burst rate for all subjects across groups and sessions.

The model comparison showed evidence for an effect of Group (BF = 10.4) but gave evidence against an effect of Session (BF = 0.064) and gave evidence against interaction between Group and Session (BF = 0.23).

The Parkinson’s patients had 5–17% (median: 11%) lower rate in the non-medicated state compared to healthy controls (*P* < 0.001). In the medicated state, the patients had between 13% less to 1% higher (median: 6% less) bursts rate than healthy controls (*P* = 0.06). The change in burst rate in the patients from after taking the medication varied from a 4% reduction to 8% increase (median 2% increase) and did not significantly differ from zero (*P* = 0.60). The healthy controls showed a change in burst rate from the first to the second that ranged from a 9% decrease to a 2% increase (median: 3% decrease). The change in burst rate between session for the healthy controls was not significantly different from zero (*P* = 0.22).

### Burst duration

The beta bursts had a median duration between 73 and 75 ms in both sessions and groups (see Table 3), and 95% of the durations were within 35–170 ms. The median duration of the beta bursts corresponded roughly to a single oscillatory cycle in the beta frequency range at ∼13–14 Hz.

**Table 3:** Group-level summary of beta burst features (medians and 95%-predictive intervals)

Group-session	Bursts/min	Duration	Inter-burst interval	dSPM peak amplitude
Parkinson’s patients 1/non-medicated	106 (86–127)	75 ms (37–169)	199 ms (11–3805)	0.99 (0.62–1.73)
Parkinson’s patients 2/medicated	108 (88–130)	75 ms (37–168)	168 ms (9–3470)	1.01 (0.62–1.75)
Healthy controls 1	120 (98–142)	73 ms (36–162)	136 ms (7–2844)	0.95 (0.59–1.65)
Healthy controls 2	116 (94–138)	73 ms (35–164)	148 ms (7–3137)	0.98 (0.61–1.68)

dSPM = dynamic statistical parametric mapping.

The comparison of the burst durations showed evidence against an effect of Session (BF = 0.047), gave evidence against an effect of Group (BF = 0.18) and gave evidence against the interaction between Session and Group, though the evidence is in the inconclusive range (BF = 0.57).

### Inter-burst intervals

**Figure 4:**
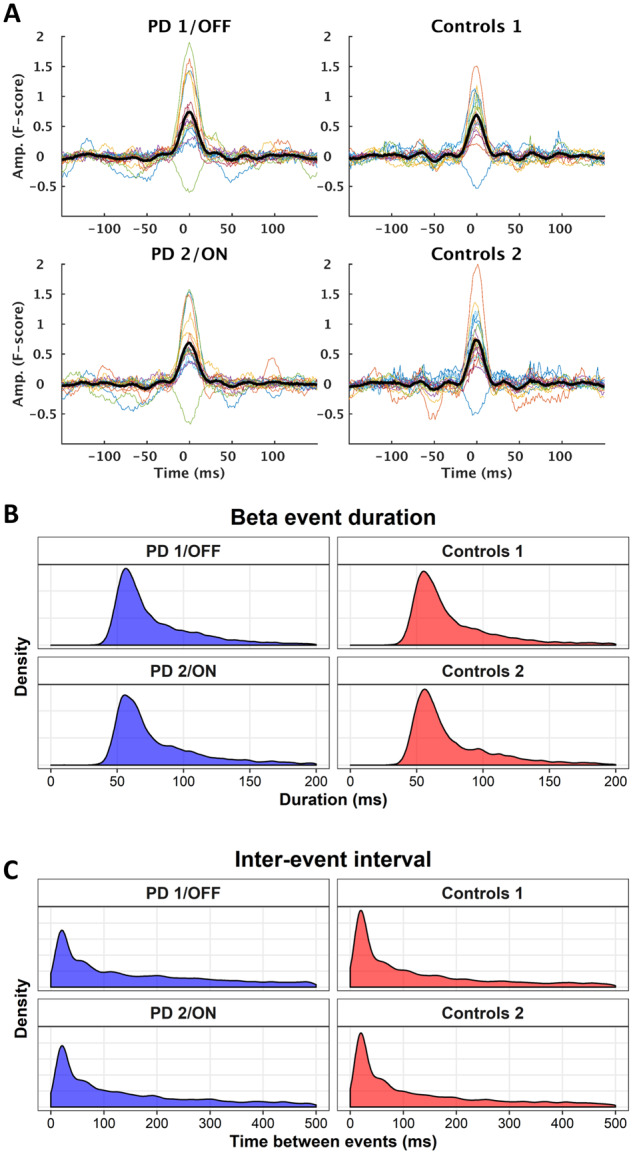
**Beta burst features.** **(A**) Average beta bursts time-locked to the burst peak for each group/session. Thick lines are the grand average, and coloured lines are individual subjects. Pooled distributions of the burst duration (**B**) and inter-burst intervals (**C**) across groups and sessions.

The inter-burst intervals had a skewed distribution with a high probability of short intervals below 200 ms with few longer intervals that could last up to seconds (Fig. 4B). The model comparison showed evidence against an effect of Session (BF = 0.050) and strong evidence both for an effect of Group (BF = 294) and for an interaction between Group and Session (BF = 5141).

The model showed a median inter-burst interval of 199 ms (mean: 653 ms, 95%-CI: 11–3805 ms) for patients off medication, compared to a median inter-burst interval of 136 ms (mean: 461 ms, 95%-CI: 7–2844 ms) for healthy controls in the first session (*P* < 10^−4^). The median inter-burst interval decreased to 168 ms (mean: 560 ms, 95%-CI: 9–3470 ms) on medication, corresponding to a 10% decrease (CI: 4–14%; *P* = 2 × 10^−4^). The inter-burst interval changed in the opposite for the healthy controls and increased by 8% (CI: 3–14%) between sessions (*P* = 0.004).

### Peak amplitude

Figure 4A depicts averaged beta bursts time-locked to the peak amplitude. The peak amplitude of the beta bursts only differed between sessions, independent of the group. The model comparison of the peak amplitude showed evidence for an effect of Session (BF = 1.7 × 10^9^), but evidence against an effect of Group (BF = 0.47), and evidence against the model that included the interaction between Session and Group (BF = 0.42)—though the BFs are in the inconclusive range for the two latter model comparisons. The peak amplitude increased for both controls and patients in the second session; with an increase of 4% (CI: 3–5%; *P* < 10^−4^) for controls and an increase of 2% (CI: 1–3%; *P* = 0.002) for the Parkinson’s patients.

### Sensitivity and specificity analysis

**Figure 5:**
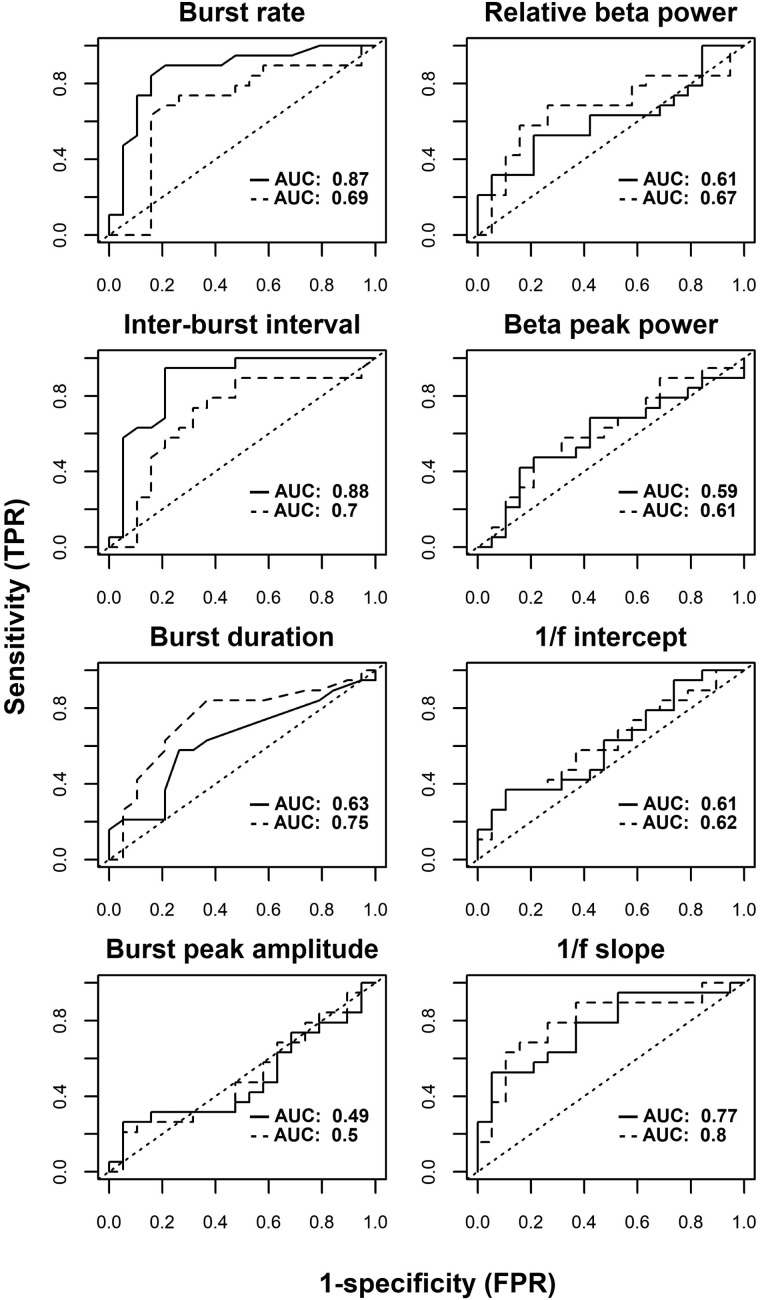
**Receiver operating characteristic (ROC) curves.** The insert text is the area under the curve for the first session (solid lines) and the second session (dashed lines). AUC = area under the curve; TPR = true positive rate; FPR = false positive rate.

The ROC curves for classification of patients or controls based on the various features of beta bursts and quantifications of the PSD is shown in Fig. 5. The highest rate for correctly discriminating between groups was on the burst rate and the inter-burst interval with an area under the ROC curve of 0.87 and 0.88 in the first session (Table 4). The burst duration was only at 0.60 and peak amplitude was at baseline probability. For quantifications of the PSD, the *relative beta power* and *beta peak power* were both around 0.60. The slope of the 1/f log-linear fit performed the best of the PSD measurements at 0.77 for the first session/non-medicated.

**Table 4:** Sensitivity/specificity of the summary measures of beta-band activity

Measurement	AUROC		Optimal threshold	
	Session 1	Session 2	Session 1	Session 2
Relative beta power	0.61	0.67	0.34	0.36
1/f intercept	0.61	0.62	−1.87	−1.89
1/f slope	0.77	**0.80**	0.82	0.73
Beta peak power	0.59	0.61	0.39	0.36
Burst rate	**0.87**	0.69	115	105
Burst duration	0.63	0.75	67	66
Inter-burst interval	**0.88**	0.70	164	178
Peak amplitude	0.48	0.50	1.59	1.66

The area under the ROC curve and the optimal numerical threshold that gave the highest performance in separating the two groups for each measurement. Bold values signify the highest AUROCs for each session.

AUROC = area under the receiver-operator characteristic curve.

For the second session/medicated, the intercept of the lognormal regression performed the best of all the measurements at 0.80. When the patients were on medication, all other measurements dropped below 0.70 in the analysis of the second session/medicated.

### Comparison across thresholds

**Figure 6:**
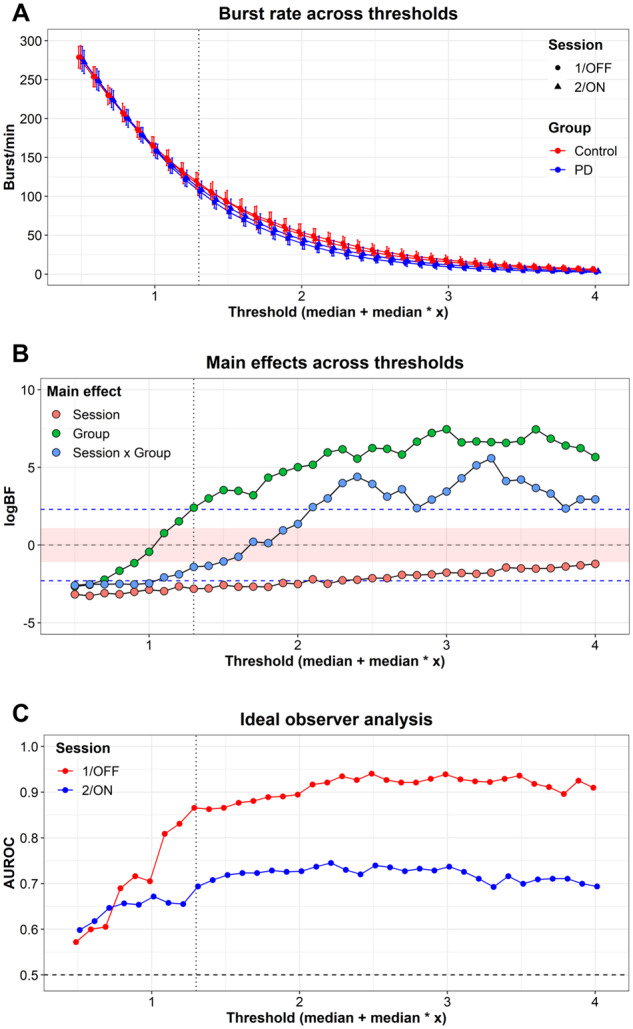
**Comparison across thresholds for defining beta bursts.** (**A**) The beta burst rate across thresholds used to define beta bursts for both groups and sessions. (**B**) The results of the Bayesian-model comparison across thresholds. The red area indicates the interval where the BFs are considered ‘inconclusive evidence’, and the dashed red lines indicate ‘substantial evidence’ for (upper line) or against (lower line) the alternative hypothesis, following the guidelines by Wetzels *et al.* (2011). (**C**) Ideal observer analysis showing the area under the ROC curves for detecting Parkinson’s patients from healthy controls based on burst rate across thresholds. The vertical dashed lines indicate the threshold used in the primary analysis. *logBF* = logarithm of Bayes factor; AUROC = area under the receiver-operator curve.

To investigate how the threshold for defining beta bursts influenced the inference, we repeated the comparison of the burst rate across a range of thresholds. Figure 6B shows the BFs of the comparison across the thresholds. The model comparisons for all thresholds above one unit of medians favoured a difference in the number of beta bursts between controls and patients with the patients having fewer beta bursts than the controls additional ROC curves for the mean and mode is presented in the supplementary material. At higher thresholds, the comparison favoured an interaction between Group and Session, with an increase in the burst rate after taking dopaminergic medication but also increased variation (Fig. 6A). Since the inference one would draw at different thresholds is consistent across thresholds (with the exception of the very low and high thresholds), we conclude that the inference is not overtly dependent on the precise numerical threshold used to define beta bursts to make the inference in the main analysis invalid.

The sensitivity/specificity analysis to discriminate between patients and controls across thresholds were similar consistent across thresholds as in the main analysis (Fig. 6C). At lower threshold (<1), there were less precise classification between groups for both session 1/non-medicated and session 2/medicated. The area under the ROC curve was consistent across higher thresholds between 85–95% in the first session and 70–75% in the second session.

### Beta burst rate and motor symptoms

**Figure 7:**
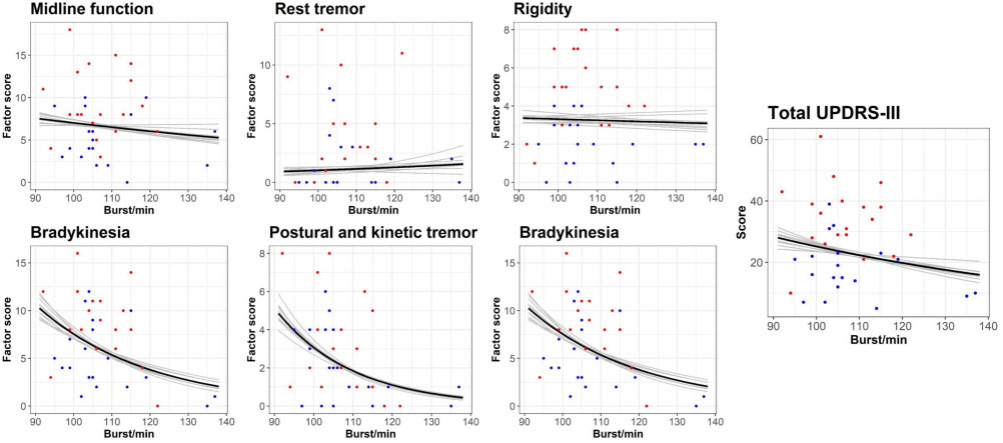
**Relation between the beta burst rate and MDS-UPDRS-III subscales.** Coloured dots are individual measurements off medication (red) and on medication (blue). The solid line is the regression model of burst rate on the score on MDS-UPDRS-III subscales. The shaded lines are on k-fold cross-validation of the regression models. MDS-UPDRS-III = Movement Disorder Society’s Unified Parkinson’s Disease Rating Scale, part III.

Figure 7 shows the marginal predicted effects of the burst rate and the subscales of the MDS-UPDRS-III from the regression models. The burst rate scaled negatively with bradykinesia (*P* = 0.006). The regression model predicted a decrease in bradykinesia rating of 28% (95%CI: 9–46%) when the burst rate increased by 10. The burst rate further scaled negatively with postural/kinetic tremor (*P* = 0.003), predicting 40% (95%CI: 16–59%) decrease in symptom rating when the burst rate increased by 10. We saw no evidence that midline function (*P* = 0.46), rest tremor (*P* = 0.75), rigidity (*P* = 0.88) nor lower limb bradykinesia (*P* = 0.22) scaled with the burst rate.

## Discussion

The primary aim of this study was to explore whether beta burst characteristics, recorded with MEG during a 3-min period at rest with eyes open, differed between Parkinson’s patients and healthy controls. As a secondary aim, we also explored whether beta burst characteristics vary with the Parkinson patients’ dopaminergic medication. Finally, as a third aim, we explored whether beta burst rate was related to symptom severity in Parkinson’s disease.

Parkinson’s patients (off medication) showed a 5–17% lower beta burst rate compared to healthy controls. Neither the duration nor the amplitude of the beta bursts differed between patients and controls. The consistency in duration and amplitude suggests that the mechanisms that generate the cortical beta bursts are preserved in Parkinson’s disease and that it is primarily the *burst rate* that underlies the disease-related changes in cortical beta power often reported for Parkinson’s disease.

We did not find overwhelming evidence for a modulation of burst rate by dopaminergic medication. Since the study was exploratory and we did not have prior expected effect size of medication and that our sample size was relatively small (*N* = 19), there might be effects of medication that we have not detected with this analysis approach. Worth noting is that at higher thresholds for determining beta bursts (Fig. 7B), there was evidence for an effect of medication on the burst rate. Most notably, the inter-burst interval showed was effected by medication. When the Parkinson’s patients were on medication, the distribution of the inter-bursts interval changed to resemble the distributions of the healthy controls. What this means in terms of disease-related mechanisms is currently unclear, as the underlying dynamics that drive the beta bursts are insufficiently understood. Speculatively, it is possible that the shift in the inter-burst interval following dopaminergic medication is driven by a change in the distal drive from dopamine modulated activity in basal ganglia or thalamus. However, more research is needed to understand how the cortical beta bursts are driven by deeper sources, which directions the connection goes, and how this is modulated by dopaminergic medication.

These results are in line with the research from Sherman *et al.* (2016), who proposed that beta bursts in the cortex is caused by a short distal drive to the upper laminar layers lasting around 50 ms, in combination with a sustained excitatory proximal drive between the upper and lower cortical layers. The reduction in spontaneous cortical beta bursts could be driven by a reduction in distal connections from thalamus or basal ganglia leading to a reduction in the beta burst rate. This might mean that the affected mechanism leading to reduced activity in the cortical beta band in Parkinson’s disease is the distal drive that initiates the beta bursts rather than alterations in the cortical generators themselves. The distal input to the cortex could stem from structures in the midbrain affected by the loss of dopamine in Parkinson’s disease. Modulation of the dynamic changes in the beta activity due to the dopaminergic medication has been shown in deep brain recordings from STN in Parkinson’s patients (Tinkhauser *et al.*, 2017*a*, *b*).

The decrease in beta burst rate was associated with an increase in symptom severity for bradykinesia and postural/kinetic tremor in the Parkinson’s patients. Such a link between burst rate and bradykinesia is in line with previous studies showing that decreased beta power in the cortex is related to increased bradykinesia (Airaksinen *et al.*, 2012, 2015; Melgari *et al.*, 2014).

Our results show a disease-related reduction in beta burst rate in Parkinson’s patients compared to healthy control and related to the manifestation of motor symptoms. This reduction of beta burst rate should translate into changes in beta band PSD when data are analysed using Fourier-based methods: a reduction in the beta band PSD is compatible with the reduction in the number of spontaneous beta bursts. However, in our results, we did not observe any conclusive differences in beta band PSD between Parkinson’s patients and healthy controls that correspond to those we report for the analysis of beta bursts (only an inconclusive trend for the relative beta power). Importantly, frequency-domain analysis of the beta band, using the traditional Fourier-transform method, was less sensitive for picking up statistically meaningful differences in beta activity between Parkinson’s patients and healthy controls, as compared to an analysis based the core features of the underlying beta bursts. The sensitivity/specificity analysis showed that burst rate and inter-burst intervals performed best in separating healthy controls from Parkinson’s patients off medication. The sensitivity/specificity of the beta burst features dropped when the patients were on medication. However, the general slope of the broadband PSD increased in sensitivity/specificity in separating Parkinson’s patients when the patients were on dopaminergic medication.

Though beta burst rate was the most sensitive in separating Parkinson’s patients from controls, it is worth noting that there was variation between the sessions for the healthy controls. The variation may reflect the test–retest variability of the measurements that, in this case, were between a 9% decrease to a 2% increase in beta burst rate. This variation could also reflect a circadian effect on the spontaneous beta bursts. It has previously been shown that the frequency domain beta power varies with the time of the day (Wilson *et al.*, 2014). It is plausible that similar circadian effects apply to beta bursts in the time-domain. All participants—Parkinson’s patients and controls alike—were tested in the morning and again before noon on the same day in our study. It is therefore interesting that the direction of the change from session 1/non-medicated to session 2/medicated were in opposite directions: a trend towards decreased burst rate for healthy controls and a trend towards increased burst rate for the Parkinson’s patients. If the change in burst rate for the healthy controls signifies a circadian effect, we can speculate that such decrease in burst rate would interfere with an increase in burst rate in the patients to mask the medication effect. To answer this question, we need more research into the short-term circadian changes in beta burst rate.

The literature on Parkinson’s disease and cortical beta band activity suggests that the presence of cortical beta band activity is inversely related to motor function: a decrease in beta band activity indicates an increased sensitivity to efferent and afferent sensorimotor signals, whereas increased activity has been linked to inhibition of sensorimotor signals (Brown, 2007; Engel and Fries, 2010). Indeed, close temporal proximity between beta bursts and go cues leads to longer reaction times (Little *et al.*, 2019; Lofredi *et al.*, 2019) and less likelihood of detecting sensory stimuli close to the sensory threshold (Shin *et al.*, 2017), showing that the proximity of beta bursts hinders immediate sensorimotor processing. Spontaneous beta bursts thus seem to have a transient inhibitory effect on the sensorimotor processing, but might at the same time serve as a signal that is necessary to maintain an optimal state of sensorimotor processing (Engel and Fries, 2010; Jenkinson and Brown, 2011). This interpretation suggests that the beta bursts enable an immediate updating of the sensorimotor system by integrating the previous motor signal and proprioceptive signal (Leventhal *et al.*, 2012). The beta bursts might hence be inhibitive, as evidenced by their behavioural effects on event-related sensorimotor tasks (Shin *et al.*, 2017; Little *et al.*, 2019), but on the other hand, also serve to inform and integrate information in the sensorimotor system over a longer time. Following this reasoning, the inverse relationship between the number of spontaneous beta bursts and bradykinesia that we report in this study, might hence be due to a deficit in the integration of information in the sensorimotor system, which leads to suboptimal utilization of neural resources when initiating and performing movements manifesting as bradykinesia and kinetic tremors.

Beta band activity is altered in Parkinson’s disease, which is often evident at the frequency domain on decomposed and averaged time-series of electrophysiological activity. However, that analysis approach implicitly assumes that the average spectral signal is representative of the whole time-series. This appears not to be the case, and hence that analysis approach is not optimal. The neuronal oscillations in the beta band change over time by exhibiting transient beta bursts lasting 70–80 ms. We have shown that the beta burst amplitude and duration is similar for both healthy adults and Parkinson’s patients—but that the burst rate is reduced by more than 10% to an average of 106 bursts per minute in Parkinson’s participants, as compared to the 120 in healthy controls.

The spontaneous burst dynamics in the beta band, such as burst rate, might hold relevant information for understanding Parkinson’s disease and the neural underpinnings of disease-related motor symptoms. That the beta band is related to dopamine and altered in Parkinson’s disease can also be clearly seen in recordings of beta bursts from STN (Tinkhauser *et al.*, 2017*a*, *b*). Beta bursts are therefore a potentially useful marker of Parkinson’s disease. However, recordings of the electrical field in STN is only done in patients who undergo brain surgery and thus not feasible for diagnostic purposes. Showing that it is possible to detect differences, in the form of reduced beta burst rate, between patients and healthy controls with non-invasive MEG measurements is an important step towards better understanding of the cortical beta band and better quantification of beta-band activity, which potentially can lead to new methods for detecting and diagnosing Parkinson’s disease.

## Supplementary Material

fcaa052_Supplementary_Data

## Abbreviations

AUROC =: area under the receiver-operator characteristic curve;
BF =: Bayes factor;
dSPM =: dynamic statistical parametric mapping;
EEG =: electroencephalography;
LEDD =: levodopa equivalent daily dosage;
MEG =: magnetoencephalography;
MDS-UPDRS-III =: Movement Disorder Society’s Unified Parkinson’s Disease Rating Scale part III;
MoCA =: Montreal Cognitive Assessment;
PSD =: power spectral densities;
ROC =: receiver operating characteristic;
ROI =: region of interest;
STN =: sub-thalamic nucleus
