# Supplementary material for: Reduction of spontaneous cortical beta bursts in Parkinson’s disease is linked to symptom severity
Source: Brain Commun. 2020 Apr 28;2(1):fcaa052. doi: 10.1093/braincomms/fcaa052 (PMC7425382; doi:10.1093/braincomms/fcaa052)
Supplement: fcaa052_Supplementary_Data [file fcaa052_supplementary_data.pdf]

Supplementary material

Oscillations and 1/f in the power spectral densities

Table S1: Summary of coefficients of the 1/f regression analysis of PSD across groups and sessions (mean + s.d.). 1/f regression is the broadband log-linear regression parameters. Peaks in the beta band (13-30 Hz) alpha band (8-12 Hz) and theta band (4-8 Hz) were parametrised by fitting Gaussian functions to the PSD with the 1/f function removed. Peak frequency and peak power are estimated as the canter and height of the Gaussian functions. N is the number of participants who had a peak in the respective frequency band.

**Group-session**	**1/f regression**		**Beta**			**Alpha**			**Theta**		
	**Intercept**	**Slope**	**N**	**Peak freq.**	**Peak power**	**N**	**Peak freq.**	**Peak power**	**N**	**Peak freq.**	**Peak power**
Parkinson's patients 1/non-medicated	-2.08 (0.50)	0.81 (0.20)	19/19	18.5 (4.2)	0.36 (0.13)	14/19	9.8 (1.4)	0.33 (0.14)	6/19	7.4 (0.5)	0.27 (0.12)
Parkinson's patients 2/ medicated	-2.06 (0.51)	0.82 (0.23)	19/19	18.7 (3.4)	0.36 (0.13)	16/19	10.2 (1.4)	0.30 (0.11)	8/19	7.2 (0.6)	0.34 (0.14)
Healthy controls 1	-2.27 (0.38)	0.64 (0.14)	19/19	19.2 (3.0)	0.33 (0.15)	15/19	10.0 (1.5)	0.29 (0.13)	4/19	7.1 (06)	0.28 (0.15)
Healthy controls 2	-2.29 (0.38)	0.60 (0.17)	19/19	19.9 (4.3)	0.32 (0.13)	14/19	10.1 (1.3)	0.32 (0.17)	3/19	7.4 (0.6)	0.40 (0.20)

Table S1 provide an extended summary of the *fitting oscillations & one over f* (FOOOF) analysis (Haller et al., 2018) similar to the summary in Table 4. In addition to the 1/f log-linear regression parameters and the centre frequency and peak power of the beta band peak also presented in Table 4, Table S1 contains a summary of the centre peak frequency and peaks power in the alpha (8-13 Hz) and theta (4-8 Hz) frequency bands. About one-fourth of the participants did not show a clear peak in the alpha band (see Table S1).

Comparison with "Bayesian t-tests" (Rouder et al., 2009) across groups and session, gave evidence against a difference in alpha peak frequency (for those participants that showed a peak in the alpha band) between the patient- and healthy control groups in both the first session/non-medicated (BF = 0.36) and the second session/medicated (BF = 0.35). The same was the case for the within-group comparison across sessions in the PD group (BF = 0.35) and the within-group comparison in the

control group (BF = 0.28). Similar comparison of the peak power of the alpha peaks gave evidence against a difference between the patient- and healthy control groups in both the first session/non-medicated (BF = 0.47) and the second session/medicated (BF = 0.38), and for the within-group comparison across sessions in the PD group (BF = 0.33) and in the healthy control group (BF = 0.54).

For the theta band, only about one-in-four had a clear peak in the PSD (see Table S1). The peak frequency and peak power of the theta peaks are summarized in Table S1, for those participants who showed a theta peak. Since there were so few participants that showed theta peaks, a statistical comparison is meaningless between the groups or across sessions.

Additional sensitivity and specificity analysis

Table S2: **sensitivity/specificity of the different summary measures of beta band activity (similar to Table 4)**. The area under the ROC curve and the optimal numerical threshold that gave the highest performance in separating the two groups for each measurement. In addition to the values presented in Table 4, this table shows the values for *median*, *mean*, and *mode* of the quantification of the beta bursts. *AUROC*: area under the receiver operator characteristic curve.

**Measurement**	**AUROC**		**Optimal threshold**	
	Session 1	Session 2	Session 1	Session 2
Relative beta power	0.61	0.67	0.34	0.36
1/f intercept	0.61	0.62	-1.87	-1.89
1/f slope	0.77	0.80	0.82	0.73
Beta peak power	0.59	0.61	0.39	0.36
Burst rate	0.87	0.69	115	105
Burst duration				
Mean	0.52	0.65	81	85
Median	0.63	0.75	67	66
Mode	0.58	0.51	57	61
Inter-burst interval				
Mean	0.86	0.69	456	460
Median	0.88	0.70	164	178
Mode	0.73	0.60	19	19
Peak amplitude				
Mean	0.48	0.48	1.70	1.82
Median	0.48	0.50	1.59	1.66
Mode	0.53	0.51	1.25	1.37

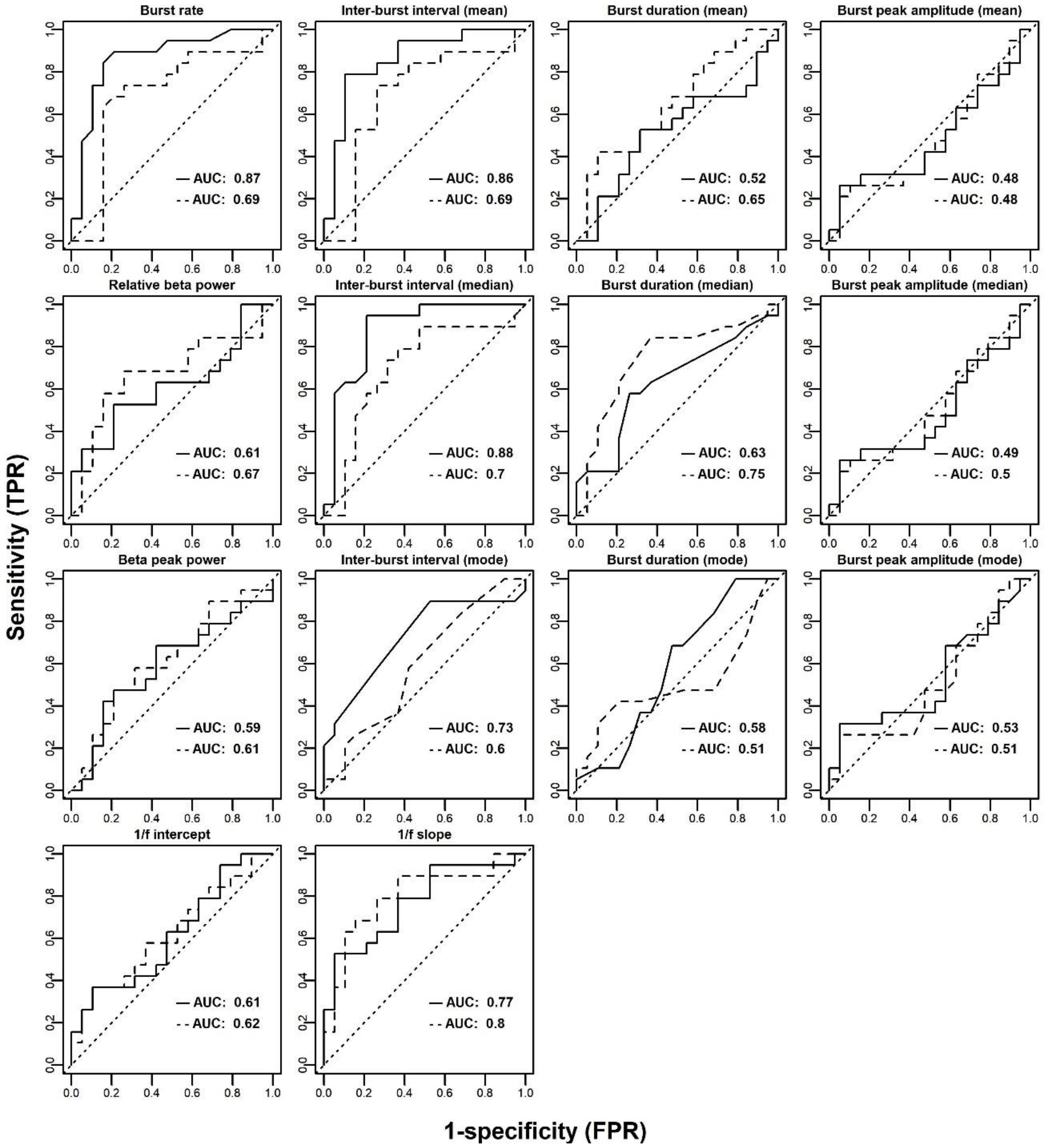

Figure S1 **receiver operating characteristic (ROC) curves (similar to Fig. 5).** The insert text is the area under the curve for the first session (solid lines) and the second session (dashed lines). In addition to the ROC curves in Fig. 5, this also displays the ROC curves for the *mode* and *mean* of *burst duration, inter-burst interval,* and *burst amplitude*. *AUC*: area under the curve. *TPR*: true positive rate, *FPR*: false positive rate.

References

Haller, M., Donoghue, T., Peterson, E., Varma, P., Sebastian, P., Gao, R., Noto, T., Knight, R. T., Shestyuk, A., & Voytek, B. (2018). Parameterizing neural power spectra. *BioRxiv*, 299859. https://doi.org/10.1101/299859

Rouder, J. N., Speckman, P. L., Sun, D., Morey, R. D., & Iverson, G. (2009). Bayesian t tests for accepting and rejecting the null hypothesis. *Psychonomic Bulletin & Review*, *16*(2), 225–237. https://doi.org/10.3758/PBR.16.2.225
